# Ecotypic differentiation of a circumpolar Arctic-alpine species at mid-latitudes: variations in the ploidy level and reproductive system of *Vaccinium vitis-idaea*

**DOI:** 10.1093/aobpla/plab015

**Published:** 2021-04-08

**Authors:** Akimi Wakui, Gaku Kudo

**Affiliations:** 1 Faculty of Environmental Earth Science, Hokkaido University, Sapporo 060-0810, Japan; 2 Botanic Gardens of Toyama, 42 Kamikutsuwada, Fuchu-Machi, Toyama 939-2713, Japan

**Keywords:** Alpine plant, low elevation, marginal population, polyploid complex, self-compatibility, *Vaccinium vitis-idaea*

## Abstract

Although plant species originated from Arctic regions commonly grow in alpine habitats at mid-latitudes, some populations of these species exist also in some specific habitats below the treeline. Local populations at lower elevations may have different origins, ploidy levels, mating systems and/or morphological traits from alpine populations, but comparative studies between alpine and low-elevation populations are scarce. We aimed to reveal the ecological and genetic differentiations between higher and lower populations of *Vaccinium vitis-idaea* in Hokkaido, northern Japan by comparing 22 populations growing in diverse environments. We analysed the ploidy level of individual populations using flow cytometry. Genetic differentiation among populations, and genetic diversity within populations were calculated using microsatellite markers. Fruit and seed production were recorded under natural conditions, and a pollination experiment was conducted to reveal the variations in mating system across populations. Furthermore, we compared shoot growth and leaf characteristics among populations. Most of the low-elevation populations were tetraploid, whereas all but one of the alpine populations were diploid. Tetraploid populations were clearly differentiated from diploid populations. Some tetraploid populations formed huge clonal patches but genetic diversity was higher in tetraploids than in diploids. Alpine diploids were self-incompatible and produced more seeds per fruit than tetraploid populations. In contrast, tetraploids showed high self-compatibility. Leaf size and foliar production were greater in tetraploid populations. Our results indicate that the genetic compositions of low-elevation tetraploid populations are different from those of alpine diploid populations. Most populations at lower elevations contained unique ecotypes suited to persistence in isolated situations. Local, low-elevation populations of typical alpine species maintain ecologically and genetically specific characteristics and could be valuable in terms of evolutionary and conservation biology. The present study demonstrates the biological importance of small and isolated populations at the edges of species distribution.

## Teaser

Plant species originated from Arctic regions commonly grow in alpine habitats at mid-latitudes, but some species exist also in specific low-elevation habitats. We investigated ecological and genetic differentiations between alpine and low-elevation populations of *Vaccinium vitis-idaea* in northern Japan. Most of the alpine populations were diploids as well as northern populations. Unexpectedly, most of the low-elevation populations were tetraploids that were first record in this species. Tetraploids showed unique genetic composition, higher self-compatibility and larger morphological traits compared to diploids. Therefore, alpine and low-elevation populations are composed of different ecotypes suited to persistence in each specific environment.

## Introduction

Distribution patterns of many northern species reflect the migratory history through glacial–interglacial cycles in the Pleistocene (Hewitt 2004). Recent phylogeographic studies have provided remarkable insights into the migration pattern, adaptation and species differentiation of Arctic-alpine plants, and emphasized the importance of marginal populations located at the southern edges of the distribution range (e.g. Alsos *et al.* 2005; Winkler *et al.* 2012; Eidesen *et al.* 2013). Stranded populations at mid-latitudes are usually isolated from one another because habitats that are suitable for a given species tend to exist quite locally and sparsely near the distribution edges (Hampe and Petit 2005). Several studies reported that isolated populations in mid-latitudinal alpine areas often possess unique haplotypes, different from those of corresponding Arctic populations (DeChaine and Martin 2005; Schönswetter *et al.* 2005; Ikeda *et al*. 2008, 2009). Therefore, comparisons of alpine populations along a latitudinal gradient provide us clues about the migratory history and subsequent adaptation process of plants that originated in Arctic regions.

Marginal populations often show enhanced potential to adapt to local environmental conditions (Abeli *et al.* 2014). From a population genetic point of view, the appearance of new alleles or the fixation of advantageous mutations is especially beneficial in isolated populations (Kawecki 2008). Small population size and/or a lack of effective pollinators often results in high level of inbreeding or self-pollination in isolated populations (Arnaud-Haond *et al.* 2006; Mimura and Aitken 2007). Consequently, selfing ability and moderation of inbreeding depression may have evolved in isolated populations (Mee and Moore 2014). Furthermore, there are several studies reporting the occurrence of polyploid populations at the marginal parts of the whole distribution area (Rivero-Guerra 2008; Karunarathne *et al.* 2018; Kudo and Hirao 2020). High ploidy level can contribute to maintain genetic variability and reduce inbreeding depression (Rosche *et al.* 2016; Rosche *et al.* 2017; Siopa *et al.* 2020), and improve the performance of vegetative properties (Otto 2007), thus polyploidization may facilitate the maintenance of isolated populations and accelerate local adaptation (Abeli *et al.* 2014). Although there are some studies focusing on the ecological characteristics of small and isolated marginal populations (e.g. Giménez-Benavides *et al.* 2007; Yakimowski and Eckert 2007), our general understanding of local adaptation in marginal populations is still limited.

**Figure 1.**
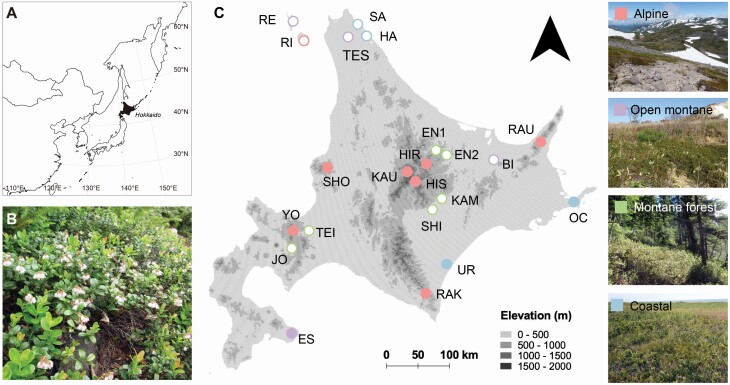
(A) Location of Hokkaido, Japan. (B) *Vaccinium vitis-idaea* in flowering season. (C) Distribution of 22 study populations in Hokkaido and pictures of four habitat types. Red circles represent alpine habitat, purple circles represent open-montane habitat, green circles represent montane-forest habitat and blue circles represent coastal habitat. Diploid populations are represented as filled circles and tetraploid populations are represented as open circles. Population IDs correspond to those listed in Table 1.

Although plant species originated from Arctic regions usually grow in alpine zones above the treeline in central Japan, some populations of these species exist also at lower elevations in coniferous forests, coastal meadows and high moors in Hokkaido of northern Japan (Fig. 1A). Most of these populations show small sizes and are geographically isolated (Sato 1995; Shimizu 2004). *Vaccinium vitis-idaea* (Ericaceae) (Fig. 1B) is an evergreen dwarf shrub broadly distributed at circumpolar high latitudes (Ritchie 1955), and its southern distribution limit is located in mountainous regions at mid-latitudes (Landolt 1996). A recent study reported that low-elevation populations of this species inhabiting algific talus slopes in coniferous forests possess unique genotypes different from those of typical alpine populations within Hokkaido (Shimokawabe *et al.* 2016). This indicates that low-elevation populations are important components of genetic diversity in this species. Considering that many northern plants migrated from other continents to Japan via Hokkaido, the populations remaining in Hokkaido may be composed of diverse origins in comparison with those remaining in Honshu, the central island of Japan. Furthermore, alpine populations and low-elevation populations within Hokkaido might be derived from different origins. Genetic and ecological studies of the low-elevation populations are important for understanding the phylogeographic background of alpine plants in Japan.

The ecological characteristics of *V. vitis-idaea* at low elevations are poorly understood. The climatic conditions of its low-elevation habitats are very different from those of its alpine habitats, and individual populations are generally small and isolated from one another. The species is reported to be partially self-incompatible (Guillaume and Jacquemart 1999). For survival and maintenance of local populations under such conditions, however, low-elevation populations might have adapted to the local environments differently from alpine populations and therefore may possess unique genetic structures, morphological traits and mating systems.

In the present study, we investigated the ploidy level, genetic structure, mating system and ecological characteristics of 22 *V. vitis-idaea* populations across various environments in Hokkaido, aiming to reveal the genetic and ecological characteristics of isolated populations in low-elevation habitats compared to those in typical alpine habitats. Specifically, we attempt to answer the following questions: (i) Are alpine populations genetically different from those located at low elevation? (ii) Do they show different morphological traits or mating systems? We hypothesize that the low-elevation populations show unique genetic signatures due to local adaptation, small population sizes and isolation, and that the low-elevation populations may be maintained by selfing and/or morphological adaptation under environmental conditions specific to low elevations.

## Materials and Methods

### Study species and study area

*Vaccinium vitis-idaea* is an evergreen dwarf shrub (5–30 cm in height) with creeping stems (Ritchie 1955) that sometimes expands via stolons to form large clonal patches (>30 m in diameter) (Persson and Gustavsson 2001). This species is broadly distributed in boreal forests and bogs across the northern hemisphere (Garkava-Gustavsson *et al.* 2005), and it mainly inhabits alpine regions at mid-latitudes. In Hokkaido, it grows mainly in alpine zone but also exists at lower elevations in unique environments, such as algific talus slopes and coastal grasslands. Flowering occurs in early summer, the flowers are pollinated by bees, and the fruits are dispersed by animals (Ritchie 1955). Partial self-incompatibility leads to reduced numbers of developed seeds due to early acting inbreeding depression (Guillaume and Jacquemart 1999). This species is commonly known to be diploid (2*n* = 2*x* = 24, Ritchie 1955), including Japanese alpine populations (Shimizu 1982). Based on genetic analyses by Ikeda *et al.* (2015), *V. vitis-idaea* populations in Japan are thought to have persisted since before the last glacial period.

From 2016 to 2019, we conducted field surveys of 22 *V. vitis-idaea* populations in Hokkaido (Table 1; Fig. 1C). These populations were classified into four habitat types based on their elevation and growing environment as follows: alpine habitat (typical alpine environment above the treeline: 1400–2000 m elevation, eight populations), open-montane habitat (grassland in the montane zone: 300–900 m, four populations), montane-forest habitat (algific talus slopes surrounded by coniferous forests: 300–900 m, six populations) and coastal habitat (coastal grassland or moors: 0–100 m, four populations). Generally, alpine populations are large and continuously distributed in each mountain area, while most montane and lowland populations are small and isolated from one another (see estimated population size in Table 1).

**Table 1.** Geographic details and genetic characteristics of 22 populations studied, and mean ± standard deviation in each of diploid and tetraploid populations. See Fig. 1 for locations in Hokkaido. Ploidy: 2*x* = diploid, 4*x* = tetraploid; Size: estimated population size as three size classes (S = smaller than 1000 m^2^, M = 1000–10 000 m^2^, L = larger than 10 000 m^2^); *N*: number of sampled ramets; Genet: number of genets; *P*_A_: number of private alleles; *A*_r_: allelic richness; *H*_e_: expected heterozygosity; *H*_o_: observed heterozygosity; 1 − *t*_m_: multilocus selfing rate.

ID	Locality	Habitat type	Ploidy	Size	Latitude (N)	Longitude (E)	Elevation (m)	*N*	Genet	*P*_A_	*A*_r_	*H*_e_	*H*_o_	1 − *t*_m_
KAU	Mt. Kaun	Alpine	2*x*	L	43°33′27	142°51′44	1907	30	14	0	1.29	0.29	0.25	0
HIS	Hisago lake	Alpine	2*x*	L	43°32′39	142°52′24	1671	30	16	0	1.32	0.32	0.30	0.08
HIR	Mt. Hira	Alpine	2*x*	L	43°45′40	143°00′28	1721	30	15	1	1.31	0.31	0.28	0
YO	Mt. Yoich	Alpine	2*x*	M	43°01′58	141°01′21	1485	25	5	0	1.27	0.27	0.18	0.24
RAU	Mt. Rausu	Alpine	2*x*	L	44°05′03	145°07′50	1445	25	8	0	1.27	0.27	0.20	0.08
SHO	Mt. Shokanbetsu	Alpine	2*x*	L	43°43′10	141°31′31	1468	30	22	1	1.35	0.35	0.26	0.06
RAK	Mt. Rakko	Alpine	2*x*	M	42°16′21	143°06′40	1471	30	14	0	1.38	0.38	0.33	0
ES	Mt. Esan	Open-montane	2*x*	M	41°48′35	141°09′26	352	30	24	1	1.34	0.34	0.32	0.05
UR	Urahoro	Coastal	2*x*	M	42°43′08	143°41′49	1	30	13	0	1.29	0.29	0.24	0.30
OC	Ochiishi	Coastal	2*x*	S	43°11′50	145°31′05	62	25	7	0	1.29	0.29	0.32	0
Mean ± SD in 2*x*										0.3 ± 0.48	1.31 ± 0.04	0.31 ± 0.04	0.26 ± 0.05	0.08 ± 0.11
RI	Mt. Rishiri	Alpine	4*x*	M	45°10′51	141°14′27	1692	30	9	0	1.46	0.49	0.44	0.30
BI	Bihoro	Open-montane	4*x*	S	43°38′58	144°15′05	525	25	8	0	1.33	0.38	0.48	0.19
TES	Teshio	Open-montane	4*x*	S	44°59′15	142°08′26	429	30	3	1	1.36	0.42	0.54	—
RE	Rebun	Open-montane	4*x*	S	45°22′24	141°01′14	409	30	10	2	1.39	0.43	0.39	0.29
EN1	Engaru1	Montene-forest	4*x*	S	43°55′25	143°20′37	371	25	5	0	1.43	0.47	0.60	0.41
EN2	Engaru2	Montene-forest	4*x*	S	43°53′50	143°17′09	487	25	4	0	1.53	0.54	0.63	0.37
SHI	Shikaribetsu	Montene-forest	4*x*	M	43°15′21	143°06′10	948	25	10	0	1.46	0.49	0.59	0.53
JO	Jozankei	Montene-forest	4*x*	S	42°50′24	141°11′13	720	25	5	0	1.37	0.40	0.48	0.44
TEI	Teine	Montene-forest	4*x*	S	43°40′48	141°11′48	725	25	2	0	1.32	0.35	0.38	0.28
KAM	Kamishihoro	Montene-forest	4*x*	S	43°30′37	143°11′55	828	20	1	0	—	—	—	—
HA	Hamatonbetsu	Coastal	4*x*	S	45°08′48	142°22′02	9	25	3	1	1.46	0.51	0.54	0.41
SA	Sarufutsu	Coastal	4*x*	M	45°10′53	142°19′55	8	30	29	4	1.40	0.45	0.53	0.37
Mean ± SD in 4*x*										0.67 ± 1.23	1.41 ± 0.06	0.45 ± 0.06	0.51 ± 0.08	0.36 ± 0.10

### Genetic and cytogenetic analyses

Between 2016 and 2019, we collected one leaf and a few fruits per shoot from 20 to 30 patches of *V. vitis-idaea* in each population. Sampling was performed along a 50- to 500-m transect, at 1- to 20-m intervals, depending on the population size. Because some low-elevation populations were too small to set a 50-m transect (TES, EN1, TEI, KAM, HA), sampling was performed from the entire range in these populations. Leaf samples were stored in silica gel and preserved at room temperature. Seeds were removed from fruits and stored in a freezer at −20 °C until analysis. In total, 1699 samples (600 leaves and 1099 seeds) were analysed.

DNA extraction from all leaf and seed samples was conducted using the cetyltrimethylammonium bromide (CTAB) method (Doyle and Doyle 1987). Eight microsatellite loci developed for a related species, *Vaccinium microcarpon* (vm04249, vm09532, vm48827, vm51985, vm51409, vm10462, vm01649, vm89040; Zhu *et al.* 2012), were amplified by polymerase chain reaction (PCR). We used the following PCR program: 5 min at 95 °C, followed by 30 cycles of 30 s at 95 °C, 90 s at 60 °C and 30 s at 72 °C, then a final rest for 30 min at 60 °C. Microsatellite fragments were analysed using the Applied Biosystems 3730 Genetic Analyser, and the genotypes were coded using Gene Mapper ver. 4.0 (Applied Biosystems). Gene Scan 500 LIZ Size Standard (Applied Biosystems) was used as the size standard. For leaf samples, we calculated the number of genets in all populations, treating samples with the same genotype at all loci as the same individual (genet).

**Figure 2.**
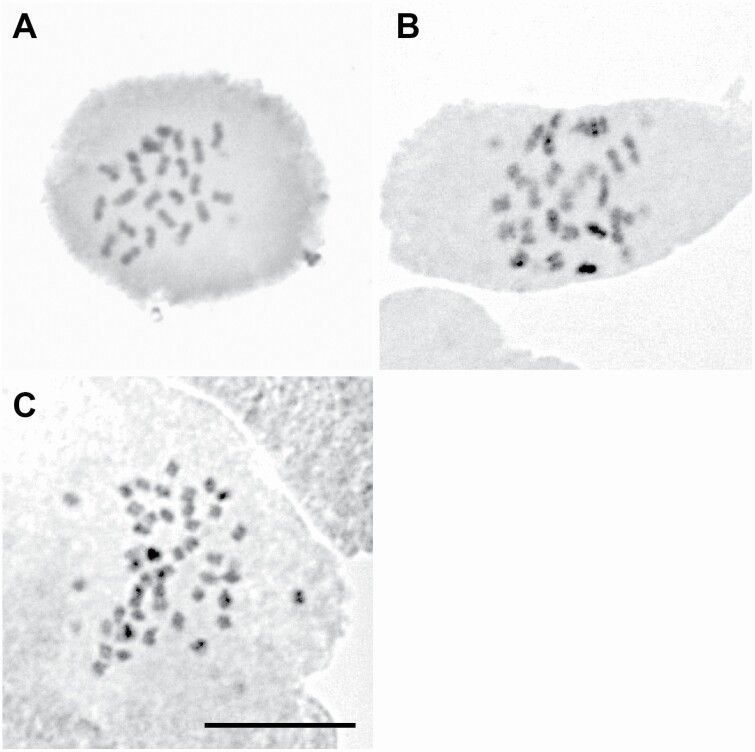
Intraspecific polyploidy in *Vaccinium vitis-idaea*. (A) KAU, 2*n* = 24 chromosomes (2*x*). (B) UR, 2*n* = 24 chromosomes (2*x*). (C) EN1, 2*n* = 48 chromosomes (4*x*). Somatic metaphase chromosomes in root tip meristems were observed on young seedlings. Bar indicates 10 µm.

Microsatellite analysis revealed that some populations showed more than two microsatellite peaks, which was unexpected for diploid species. We therefore suspected the existence of polyploid populations. To test this possibility, we conducted chromosome observation using a microscope and ploidy analysis based on amounts of DNA using a flow cytometry. Direct observation of chromosome number was conducted for three populations (KAU, UR, EN1), where KAU and UR were predicted to be diploid and EN1 was predicted to be tetraploid from the microsatellite peaks. Root tips of seedlings were pretreated with 2 mM 8-hydroxyquinoline solution for 8–9 h at 15 °C, fixed with a Farmer’s fluid for longer than 24 h at 4 °C and stained by the conventional method of acetic orcein staining-squashing. The number of chromosomes was checked under a microscope (SZH-ILLB, Olympus). In addition, DNA ploidy levels of 21 populations (all populations except for KAM) were estimated with a flow cytometry (Partec PA; Partec GmbH, Münster, Germany). Leaves for the ploidy analysis were sampled from 1 to 12 individuals in each population. We did not analyse the samples of KAM population, since we could not prepare leaf samples sufficiently fresh for the flow-cytometry analysis. Leaves were chopped in 0.2 mL of nuclei extraction buffer (CyStain UV precise P; Partec, Münster, Germany). After filtration through a 30-µm nylon mesh, crude nuclear samples were stained with 0.8 mL of 4,6-diamidino-2-phenylindole (DAPI) solution containing 10 mM Tris, 50 mM sodium citrate, 2 mM MgCl_2_, 1 % (w/v) PVP K-30, 0.1 % (v/v) Triton X-100 and 2 mg of L-1 DAPI (pH 7.5) (Mishiba *et al.* 2000) and incubated for 3 min at room temperature. The relative DNA contents of the samples were then measured. Ploidy level of target samples was estimated by calculating the ratio of relative fluorescence intensity of each sample to that of standard sample from KAU or UR population, which was confirmed to be diploid (2*n* = 24) by direct observation of chromosome number (see Fig. 2). After the identification of ploidy level in each population, we performed ANOVA comparison with ploidy level against altitude, in order to grasp the elevational distribution of each ploidy level.

Allelic richness (*A*_r_), expected heterozygosity (*H*_e_) and observed heterozygosity (*H*_o_) were calculated as genetic diversity indices using SPAGeDi 1.5a. We conducted ANOVA with ploidy levels as fixed effects to analyse differences in *A*_r_*, H*_e_ and *H*_o_ within populations, using R version 3.3.0. To examine the mating system of *V. vitis-idaea* populations, we estimated selfing rates for each population based on multilocus outcrossing rates (*t*_m_) from progeny arrays composed of genotype data of leaves and seeds from same shoots using MLTR (Ritland 2002) for diploid populations and MLTET (Ritland 1990; Murawski *et al.* 1994) for tetraploid populations. Calculations were performed with 1000 bootstraps with families as resampling units. ANOVA was conducted for selfing rate (1 − *t*_m_) between ploidies in the same way as genetic diversity indices.

We assessed genetic similarity between the populations by discriminant analysis of principal components (DAPC) to reveal the genetic structure of *V. vitis-idaea* among all populations in Hokkaido and among populations within each ploidy level. Discriminant analysis of principal components is a methodological approach used to visualize genetic similarity among populations that requires data transformation using principal component analysis (PCA) as an initial step before discriminant analysis (DA). Discriminant analysis partitions genetic variation, maximizing differences between clusters while minimizing within-cluster variation. We used Bruvo distances (Bruvo *et al.* 2004), which are particularly recommended for analysing mixed-ploidy data (Dufresne *et al.* 2014), for this analysis. All genets were treated as units of analysis. We implemented DAPC analysis in R 3.3.0 using the package poppr (Kamvar *et al.* 2014). To reveal the spatial patterns of genetic differentiation, the correlation between the *Rho* value and geographic distance (isolation by distance [IBD]; Wright 1943) was examined based on Mantel test among all populations, among diploid populations and among tetraploid populations. We obtained pairwise *Rho* values (Ronfort *et al.* 1998) as genetic differentiation indices because *Rho* statistics exhibit identical expectations for population differentiation at different ploidy levels under identical gene flow conditions (Hardy and Vekemans 2002). We calculated geographic distances as three-dimensional straight-line distances considering elevational differences using QGIS 2.8.0.

### Reproductive and morphological traits

Reproductive performance was recorded at 15 populations in 2016–17, including populations of different ploidy levels and all habitat types (Fig. 1; **see****Supporting Information—Table S1**). In early summer, one 50 m × 25 m plot was set in each population. We arbitrarily selected 50 shoots with floral buds within each plot, marked them and recorded their flower number per shoot in June and July. In August and September, we recorded the fruit number of marked shoots and calculated fruit-set rate per shoot (proportion of flowers setting fruits) for each population. One fruit per shoot was sampled from 18–25 shoots in each population and the number of developed seeds per fruit was counted **[see****Supporting Information—Table S1****]**.

Pollination experiment was conducted in five populations (Fig. 1; **see****Supporting Information—Table S1**) to reveal the variation in mating system across different habitat types and ploidy levels. In early summer, 50 individuals with floral buds were randomly chosen in each population for the experiment. One inflorescence was selected in each individual and flower number was recorded to measure fruit-set rate under natural conditions as a control. For 30 of these individuals, three more inflorescences were selected: one for a cross-pollination treatment, one for a self-pollination treatment and one for a spontaneous-selfing treatment. These inflorescences were covered with fine-meshed nylon bags before flowering to prevent insect pollination. After the flowers opened, hand pollination was conducted for the inflorescences of cross- and self-pollination treatments. For cross-pollination, pollen corrected from a different patch was deposited to the stigmas of all flowers using tweezers. Individual patches separated >40-m distance from a target plant were regarded as different individuals, referring to the previous report of clone size of *V. vitis-idaea* (Garkava-Gustavsson *et al.* 2005). Self-pollination was conducted using pollen from the same ramet. The inflorescences for the spontaneous-selfing treatment were left untreated to check for seed-set via spontaneous self-pollination. The number of treated flowers was recorded, and fruit-set rate per inflorescence was calculated in autumn. When fruits matured, if any, a single fruit per shoot was sampled to count developed seeds. At the same time, we counted unfertilized ovules and aborted seeds and calculated the seed-set rate per fruit. The mass of 30–50 developed seeds in each treatment group was measured after drying at room temperature.

Additionally, we measured floral morphology for these five populations in order to test the relationship between floral morphology and mating system. As floral morphology often reflects the mating system of individual populations, we expected that populations maintained by selfing might have less attractive floral traits in comparison with outcrossing populations (Sicard and Lenhard 2011). We randomly collected 30 flowers from different individuals in each population and measured the length and width of the corolla and the lengths of the pistil and stamen. Corolla size was expressed using the volumetric formula of an ellipsoid.

For the measurements of vegetative characteristics, 30 shoots from different individuals were chosen in each of the 22 populations, separately from individuals used for the survey of reproductive traits. For each shoot, annual shoot elongation at the end of growing season was recorded and one leaf was sampled for the measurement of leaf area and leaf mass. Leaf area was measured using the public-domain NIH ImageJ program (U.S. National Institutes of Health). Then, dry mass was measured after drying using silica gel, and leaf mass per unit area (LMA) was calculated from leaf area and dry mass.

To compare reproductive properties, we performed generalized mixed models (GLMMs) for flower number per shoot (with poisson error distribution), fruit-set rate (with negative binomial error distribution), seed number per fruit (with poisson error distribution). Two GLMMs were conducted separately for each variable, one included ploidy level as explanatory variable and the another included habitat type as explanatory variable, because there was a strong correlation between ploidy level and habitat type. Population ID and survey year were set as random effects. To compare the results of pollination experiment and measurement of floral morphology in five populations, we used generalized linear models (GLMs) and Tukey’s *post hoc* test. Fruit-set rate (with negative binomial distribution), seed number per fruit (with poisson distribution), seed-set rate per fruit (with binomial distribution) and seed weight (with gamma distribution) were compared among pollination treatments in each population. Corolla size, pistil length, stamen length (with gamma distribution, respectively), ovule number (with poisson distribution) and seed weight (with gamma distribution) were compared among populations. These analyses were conducted in R version 3.3.0, using lme4 package (Bates *et al.* 2015) for GLMMs and MASS package (Hothorn *et al.* 2008) for GLMs. For vegetative characteristics, we performed PCA, including annual shoot elongation, leaf area, leaf mass and LMA, to understand how morphological traits vary between ploidy levels and among habitat types. All the variables were standardized before the PCA. This analysis was performed using R version 3.3.0 and vegan package (Oksanen *et al.* 2015).

## Results

### Ploidy level and genetic divergence

**Figure 3.**
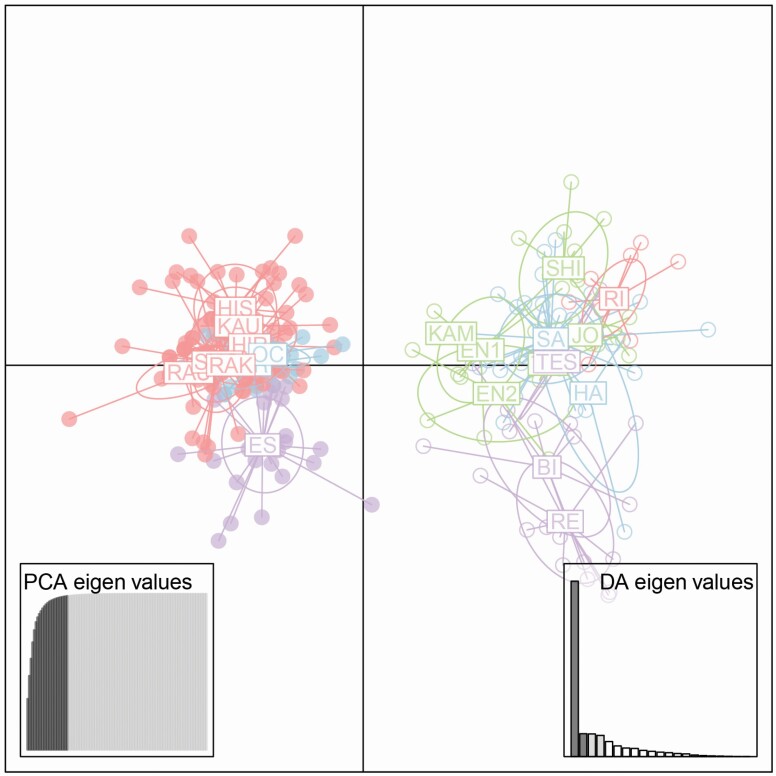
Results of discriminant analysis of principal components (DAPC). Each point represents one genotype. Diploids are represented as filled circles and tetraploids are represented as open circles. Red circles represent alpine habitat, purple circles represent open-montane habitat, green circles represent montane-forest habitat and blue circles represent coastal habitat. The bottom left graph illustrates the PCA and bottom right graph illustrates the eigenvalues of the DA.

*Vaccinium vitis-idaea* has been reported to be a diploid species with 24 chromosomes, as mentioned above. Although one or two microsatellite peaks are expected for diploid species, genetic samples from 13 populations showed three or four peaks per locus. In direct chromosome observation, individuals from KAU and UR populations showed 24 chromosomes, one or two microsatellite peaks and a relative fluorescence intensity of ≈1 when compared to a diploid standard sample (Fig. 2A and B; **see****Supporting Information—Table S2**). Individuals from EN1 population showed 48 chromosomes, three or four microsatellite peaks and a relative fluorescence intensity of ≈2 when compared to a diploid standard sample (Fig. 2C; **see****Supporting Information—Table S2**). It was revealed that all populations having one or two microsatellite peaks were estimated to be diploid (2*n* = 2*x* = 24), possessing almost the same relative DNA content as diploid populations (KAU or UR). On the other hand, all populations with more than two microsatellite peaks were estimated to be tetraploid (2*n* = 4*x* = 48), possessing almost twice as much relative DNA content as diploid populations. All samples from the same population showed the same relative DNA content, and there was no population with mixed-ploidy levels. Seven alpine populations and three low-elevation populations were recognized as diploid populations, whereas 11 low-elevation populations and one alpine population were recognized as tetraploid populations (Table 1). Although we did not check the ploidy level of KAM population by the flow cytometry, the microsatellite peaks showed similar pattern to other tetraploid populations and the genetic composition of this population was categorized in the same group as other tetraploid populations in the DAPC analysis (see Fig. 3). ANOVA analysis detected significant elevational segregation between diploids and tetraploids (*F* = 6.41, *P* = 0.02), indicating that diploids are common at higher elevations and tetraploids are common at lower elevations.

In the microsatellite analysis, we recorded a total of 54 alleles; 17 were found exclusively in the tetraploid populations, while the diploid populations had only three private alleles. At the population level, three diploid populations carried one private allele each, and four tetraploid populations carried between one and four private alleles each (Table 1). Among 600 leaf samples from all populations, 227 genets were recognized. The number of genets in the samples of 20–30 patches varied from 1 to 29 per population. Most tetraploid populations showed very few genets (Table 1) indicating that the populations are composed of small number of large clonal patches. Indices of genetic diversity (*A*_r_, *H*_e_, *H*_o_) were greater in the tetraploid populations than in the diploid populations (*F* = 14.8, 33.3, 63.7, respectively, *P* < 0.001). Multilocus estimates of selfing rate ranged from 0 to 0.53 across all populations (Table 1). Average selfing rates were significantly higher in the tetraploid populations (0.19–0.53) than in the diploid populations (0–0.30; *F* = 37.56, *P* < 0.001).

Discriminant analysis of principal components revealed a clear separation between diploid and tetraploid populations along the first axis (Fig. 3). When DAPC analysis was conducted separately in each ploidy level, tetraploid populations showed clearer genetic differentiation than diploid populations **[see****Supporting Information—Fig. S1****]**. Mantel test revealed significant IBD among all populations (*r* = 0.23, *P* = 0.03) and among the diploid populations (*r* = 0.41, *P* = 0.02), whereas there was no significant IBD among the tetraploid populations (*r* = 0.12, *P* = 0.22; **see****Supporting Information—Fig. S2**).

### Reproductive traits

The GLMM results of reproductive traits are summarized in Table 2. Flower production did not differ between ploidy levels (*P* = 0.43) and among habitat types (*P* = 0.15–0.25). Fruit-set rate was lower in the coastal habitat than in other habitats (*P* < 0.01), while there was no significant difference between ploidy levels (*P* = 0.56). Seed number per fruit significantly varied between ploidy levels and among habitat types; seed number was larger in diploids than in tetraploids (*P* < 0.001), and larger in the alpine populations than in the low-elevation populations (*P* < 0.05).

**Table 2.** Comparisons of reproductive performance (flower production per shoot, fruit-set success and seed production per fruit) between ploidy levels and among habitat types across 15 populations under natural pollination based on GLMMs. ***P < 0.001, **P < 0.01, *P < 0.05. Blank: no significance, ^#^Intercept = ploidy 2*x*, Alpine.

	Compared by ploidy					Compared by habitat type				
		Estimate	SE	*z*-value	*P*		Estimate	SE	*z*-value	*P*
Flower no./shoot	(Intercept)	2.08	0.09	24.4	<0.001***	(Intercept)	2.06	0.08	25.2	<0.001***
	Ploidy 4*x*	−0.09	0.12	−0.80	0.43	Open-montane	−0.29	0.20	−1.41	0.16
						Montane-forest	−0.17	0.12	−1.44	0.15
						Coastal	0.14	0.12	1.15	0.25
Fruit-set rate	(Intercept)	−1.45	0.30	−4.88	<0.001***	(Intercept)	−1.17	0.28	−4.14	<0.001***
	Ploidy 4*x*	−0.24	0.41	−0.58	0.56	Open-montane	−0.59	0.71	−0.84	0.40
						Montane-forest	−0.16	0.40	−0.40	0.69
						Coastal	−1.16	0.43	−2.68	0.007**
Seed no./fruit	(Intercept)	2.39	0.11	21.5	<0.001***	(Intercept)	2.41	0.16	15.4	<0.001***
	Ploidy 4*x*	−0.80	0.16	−5.18	<0.001***	Open-montane	−1.12	0.40	−2.85	0.004**
						Montane-forest	−0.73	0.22	−3.27	0.001**
						Coastal	−0.48	0.24	−2.03	0.04*

**Figure 4.**
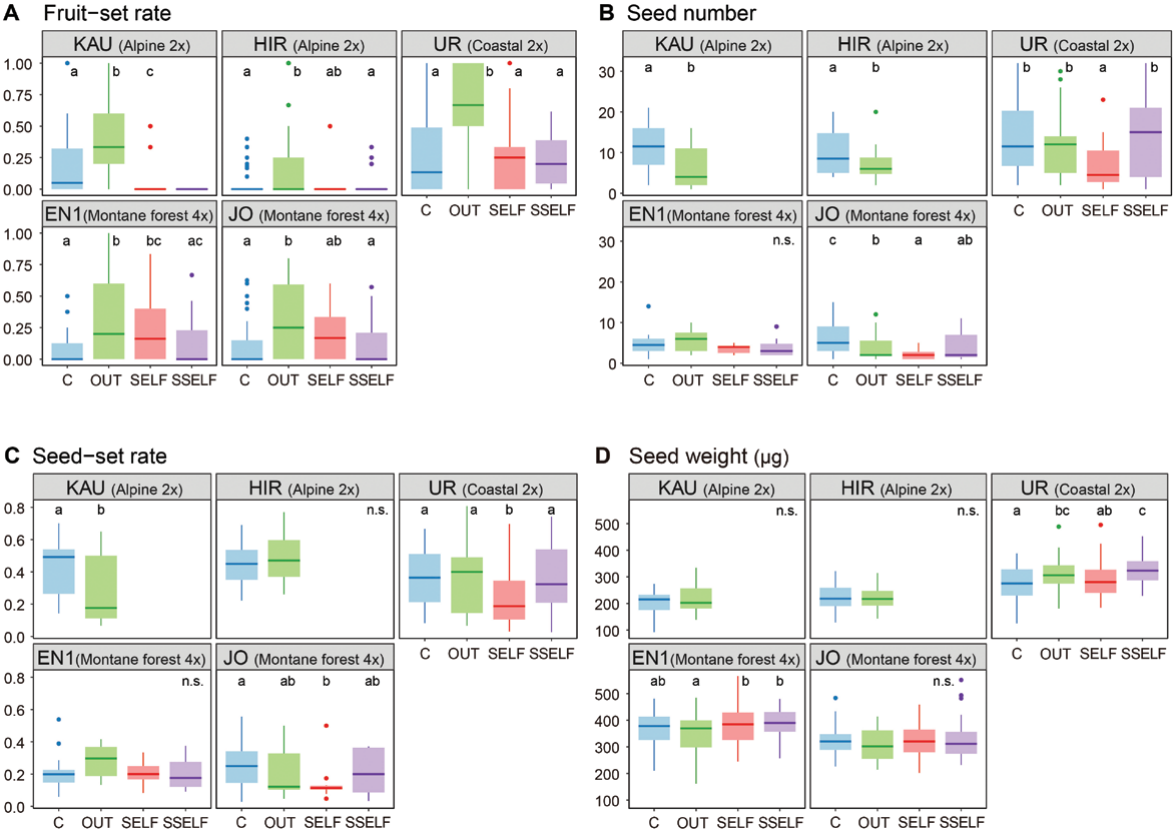
Results of pollination experiment. Comparisons of (A) fruit-set rate, (B) seed number per fruit, (C) seed-set rate and (D) seed weight (μg) across four treatments: control under natural pollination (C), cross-pollination (OUT), self-pollination (SELF) and spontaneous selfing (SSELF) in five population (KAU: Alpine 2*x*, HIR: Alpine 2*x*, UR: Coastal 2*x*, EN1: Montane-forest 4*x*, JO: Montane-forest 4*x*). Different letters represent significant differences (*P* < 0.05) according to GLM and Tukey’s *post hoc* test.

The pollination experiment revealed that the fruit-set rate of cross-pollinated inflorescences was higher than that of naturally pollinated inflorescences in all populations (Fig. 4A; **see****Supporting Information—Table S3**). This indicates that pollen limitation is common for fruit production under natural conditions. Fruit-set rates in the self-pollinated and spontaneous-selfing treatment groups varied among populations. Two diploid alpine populations, KAU and HIR, showed negligibly small fruit production on the self-pollinated and spontaneous-selfing inflorescences. In KAU, only 3 of 30 self-pollinated inflorescences produced a single fruit and none of the spontaneous-selfing inflorescences produced any fruit. In HIR, only 2 of 30 self-pollinated inflorescences produced one or two fruits and only 3 of 30 spontaneous-selfing inflorescences produced a single fruit. These results indicated that these alpine populations were mostly self-incompatible. Therefore, these treatments were excluded in the GLMs of seed-set rate, seed number and seed weight. In the coastal diploid population (UR), fruit-set rates of the self-pollinated and spontaneous-selfing inflorescences were comparable to the control, but lower than that of the cross-pollinated inflorescences. In the montane-forest tetraploid populations, EN1 and JO, fruit-set rates of the self-pollinated inflorescences were comparable to that of the cross-pollinated inflorescences, but fruit-set rates of the spontaneous-selfing inflorescences were lower than that of the cross-pollinated inflorescences.

Unlike fruit-set rate, neither seed number nor seed-set rate per fruit increased by cross-pollination (Fig. 4B and C). In UR and JO populations, fruits produced by self-pollination had fewer seed number and lower seed-set rate than fruits produced by cross-pollination, although fruits of spontaneous-selfing inflorescences produced as many seeds as fruits of cross-pollinated inflorescences. There were some variations in seed weight among pollination treatments in two of five populations, i.e., cross-pollinated and spontaneous-selfing inflorescences produced heavier seeds than naturally pollinated inflorescences in UR, and self-pollinated and spontaneous-selfing inflorescences produced heavier seeds than cross-pollinated inflorescences in EN1, although these differences were all small (Fig. 4D).

**Figure 5.**
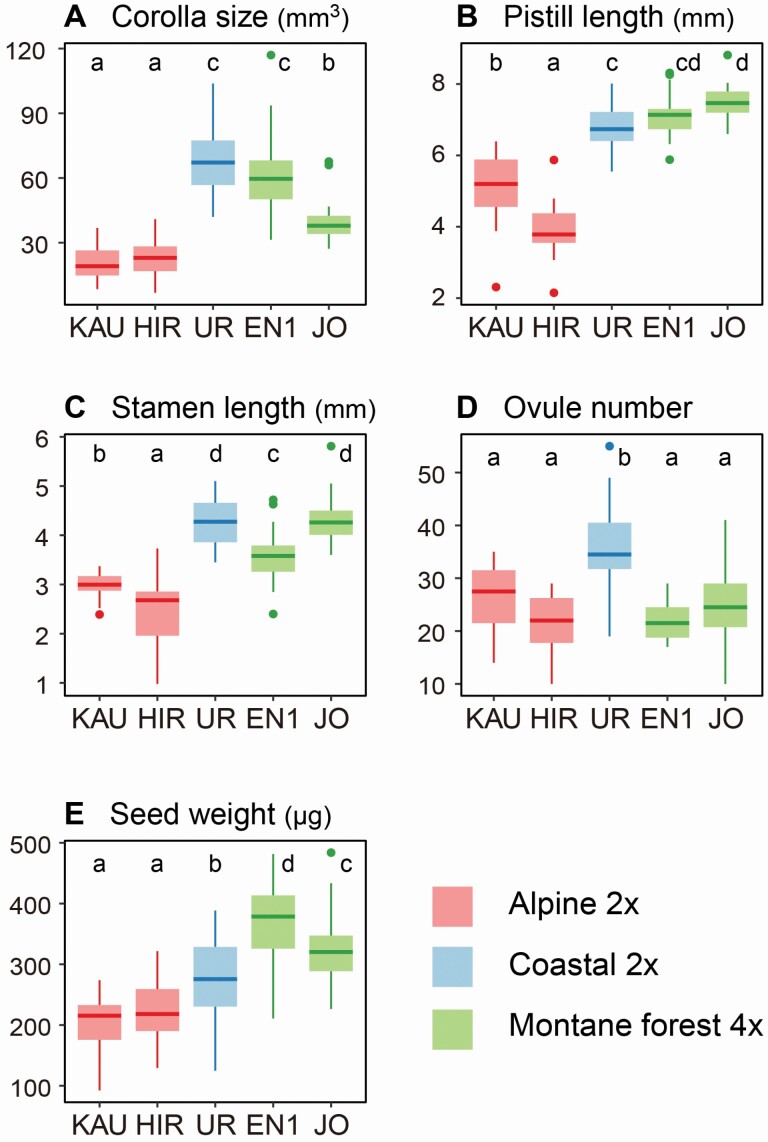
Variations in floral morphology among populations. Comparison of floral morphology among five populations (KAU: Alpine 2*x*, HIR: Alpine 2*x*, UR: Coastal 2*x*, EN1: Montane-forest 4*x*, JO: Montane-forest 4*x*): (A) corolla size, (B) pistil length, (C) stamen length, (D) ovule number and (E) seed weight. Different letters represent significant differences (*P* < 0.05) according to GLM and Tukey’s *post hoc* test.

For floral morphology, all floral traits differed between the alpine and low-elevation populations, regardless of ploidy level (*P* < 0.05; Fig. 5; **see****Supporting Information—Table S4**). Smaller corollas and shorter pistils and stamens were common in the alpine populations. Ovule number was highest in the coastal population (UR) and did not differ between the alpine (KAU, HIR) and montane-forest (EN1, JO) populations. Seed weight was greater in the low-elevation populations, especially in two tetraploid populations (EN1, JO), than in the alpine populations.

### Vegetative characteristics

**Figure 6.**
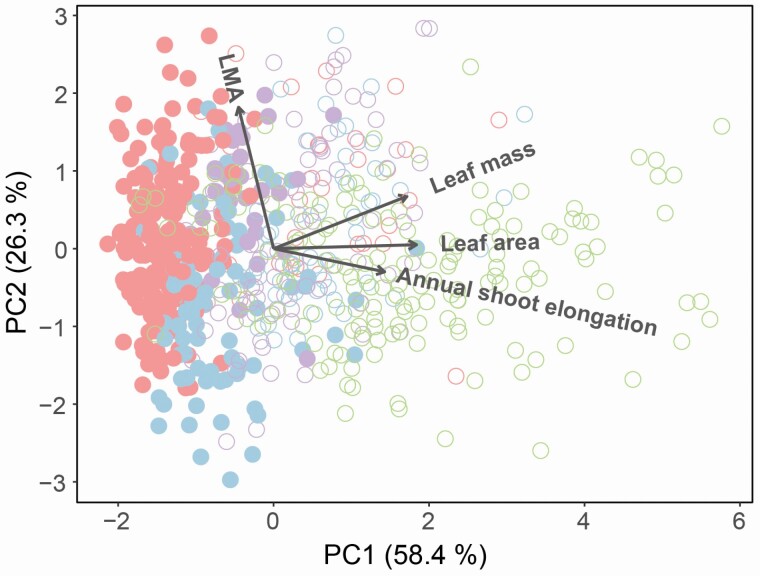
Result of PCA of vegetative characteristics of 22 populations: annual shoot elongation, leaf area, leaf mass and LMA. Red circles represent alpine habitat, purple circles represent open-montane habitat, green circles represent montane-forest habitat and blue circles represent coastal habitat. Diploid populations are represented as filled circles and tetraploid populations are represented as open circles.

In the PCA, first axis (PC1) explained 58.4 % of morphological variations and second axis (PC2) explained 26.3 % of variations. PC1 represented the differences in both ploidy level and habitat type, whereas PC2 only related with habitat type **[see****Supporting Information—Fig. S3****]**. Annual shoot length, leaf area and leaf mass were strongly related with PC1 and larger in the tetraploid populations than in the diploid populations (Fig. 6; **see****Supporting Information—Table S5**). Among the diploid populations, annual shoot length, leaf area and leaf mass were larger in the low-elevation populations (open-montane and coastal populations) than in the alpine populations. Among the tetraploid populations, the montane-forest populations showed much larger morphologies than the populations of other habitat types. Leaf mass per unit area significantly correlated with PC2 and was smaller in the montane-forest populations and the coastal populations than in the alpine populations and the open-montane populations.

## Discussion

### Ploidy variation and habitat differentiation of *Vaccinium vitis-idaea*

This is the first report of the existence of tetraploid populations of *V. vitis-idaea*. Polyploid complexes have been reported in several *Vaccinium* species (i.e. Krebs and Hancock 1989; Suda and Lysak 2001), but *V. vitis-idaea* is broadly known as a diploid species (2*n* = 2*x* = 24), with some reports of triploid (2*n* = 36) or aneuploid (2*n* = 34) populations in Finland **[see****Supporting Information—Table S6****]**. Diploids were common in alpine areas, whereas tetraploids were mostly distributed below the treeline and there were no mixed-ploidy populations detected. Even within a same mountain area, ploidy level and genetic composition differed between populations above and below the treeline. For example, HIR (alpine) and EN1 (montane-forest) were located at geographically close distance (Fig. 1), but they were diploid and tetraploid population, respectively, and classified into different genetic group in the DAPC analysis.

For the clear habitat differentiation between diploid and tetraploid populations, several reasons are considerable. First, tetraploids may have tolerance to isolated situation in terms of population persistence owing to the higher genetic diversity more than diploids (Abeli 2014). Higher level of heterozygosity is maintained in tetraploids even in isolated small populations (Rosche *et al.* 2016), resulting in lower risk of extinction (Plue *et al.* 2018). In the present study, some tetraploid populations (TES, EN1, TEI, KAM, HA) were isolated within small area (<50 m in length), and they were often composed of small number of clones. Nevertheless, higher genetic diversity was maintained, probably due to large number of alleles per locus.

Second, tetraploids might have potentially broader ecological niche being able to colonize diverse habitats at lower elevations, that are stressful environments for a cold-adapted alpine species. By the habitat segregation, tetraploids can avoid competition to diploids, that are dominant in alpine environments. Generally, polyploids have larger potential to adapt to new environments than diploids owing to the advantage of genome duplication (Soltis and Soltis 2000). Several previous studies on polyploid complex reported habitat segregation between ploidy levels (Rivero-Guerra 2008; Sonnleitner *et al.* 2010; Kudo and Hirao 2020), and some of them were related to the differences in tolerance ability to harsh environments, such as cold or drought stress (Cosendai *et al.* 2013; Thompson *et al.* 2014; Karunarathne *et al.* 2018).

Third, from the perspective of morphological adaptation, tetraploids may be advantageous at lower elevations. Compared to alpine environments, low-elevation habitats are characterized by complex vegetation structures and severe competition for light capture. Therefore, potential for morphological enlargement of tetraploids (Otto 2007) might confer selective advantages. On the other hand, there were some diploid populations at low elevations in southern and eastern parts of Hokkaido (UR, OC and ES), and there was one tetraploid population at the alpine site in northern Hokkaido (RI). The habitat of UR is a coastal grassland, that of OC is a high moor and that of ES is a sulfur wasteland on a volcanic mountain. These habitats are basically less competitive for light capture because of sparse vegetation cover or low vegetation stature, resulting in the enabled establishment of diploid populations having smaller stature. As for an absence of diploids in the alpine zone of RI, geographical background may be related. Because RI is located on a single volcano of a small island (Rishiri Island), isolated from the main land of Hokkaido, the migration of diploid ancestors might be limited or settled diploid populations might be extinct due to volcanic activity.

Furthermore, clear spatial segregation of growing habitats between diploids and tetraploids might reflect their original habitats or migration history. The origins of diploids and tetraploids in *V. vitis-idaea* are unknown, but this species is widely distributed across high latitudes of northern hemisphere, mainly in arctic tundra and boreal forests. One possibility is that diploids and tetraploids might originate in different environments or adapt to different environments through the migration process, and migrate to different elevations in Hokkaido. In the DAPC analysis, the diploid and tetraploid populations were clearly separated along the first axis. This indicates that the genetic composition of diploid and tetraploid is rather different. Another possibility is that those genetic differences might be caused by habitat differentiation or reproductive isolation after the migration to Hokkaido. Further phylogenetic study is necessary to reveal the migration history and local adaptation process in this species.

Genetic differentiation among populations was smaller in the diploid populations in comparison with the tetraploid populations **[see****Supporting Information—Fig. S1****]**. The alpine populations are generally large and continuously distributed at fellfields of alpine habitat with little snow cover. On the other hand, the low-elevation populations are often isolated and exposed to different environmental conditions in each area, as elevation and surrounding vegetation are very different among three habitat types, i.e. open-montane, montane-forest and coastal grassland. Therefore, local adaptation is expected to occur in the tetraploid populations more frequently than in the alpine diploid populations. Furthermore, increased genome size and frequent selfing in tetraploids may allow faster accumulation of mutations and contribute to accelerate local adaptation (Abeli *et al.* 2014). In addition, the lack of significant IBD in the tetraploid populations **[see****Supporting Information—Fig. S2****]** might be because of isolated distributions accompanied with many geographical barriers between populations.

### Maintenance mechanism of isolated populations

There were no differences in flower and fruit production between ploidy levels and habitat types, except for reduction of fruit-set rate in the coastal habitat. This indicates that reproductive activity is maintained even in isolated populations at lower elevations. On the other hand, seed number per fruit was significantly lower in the tetraploid populations than in the diploid populations, and lower in the low-elevation populations than in the alpine populations. Because there was little difference in ovule number per flower between diploid and tetraploid populations (Fig. 5D), small seed production in tetraploids is due to lower seed-set rate; this may be related to the general trend of lower fertility in tetraploids compared to diploids (Ramsey and Schemske 2002).

The mating system differed between the ploidy levels. Tetraploids were self-compatible, whereas most diploids were self-incompatible. Furthermore, spontaneous selfing was observed in the tetraploid populations. The evolution of selfing in isolated populations has been predicted (Mee and Moore 2014) and reported in previous studies (Arnaud-Haond *et al.* 2006; Rosche *et al.* 2017). There are some explanations for the evolution of selfing in tetraploid populations. One explanation is a reproductive assurance. Small, isolated populations might have experienced a strong bottleneck in the past, resulting in lower genetic diversity (Kawecki and Ebert 2004). Actually, there were several tetraploid populations in which only a few large genets dominated. In this situation, opportunities for outcrossing are restricted, and selfing ability is advantageous for reproductive assurance. Since tetraploids have twice as many alleles than diploids, homozygosity of deleterious alleles rarely occurs even with repeated selfing or inbreeding. Thus, tetraploids can compensate for the genetic load caused by selfing with high allele diversity and heterozygosity (Soltis and Soltis 2000). On the other hand, one diploid population in the coastal habitat (UR) also had high selfing ability. Although this population was relatively large, it was strictly isolated from the other diploid populations. Therefore, it might have experienced a bottleneck in the past and overcome self-incompatibility as a result.

The other explanation is linked to whole-genome duplication. Considering that tetraploids originate within diploid populations, tetraploids should be at a reproductive disadvantage compared to diploids in the early stage. Being the minority cytotype in the population, tetraploids are expected to be hard to reproduce by cross-pollination, which would lead to extinction from the population, i.e. minority cytotype exclusion hypothesis (MCE; Levin 1975). Thus, evolution of self-fertility could be a mechanism that counteract the MCE allowing new tetraploids to establish within a diploid population. The collapse of self-incompatibility and the mitigation of inbreeding depression are often caused by polyploidization itself, and contribute to the establishment of polyploid population at initial colonization stage (Siopa *et al.* 2020).

Seed number and seed-set rate per fruit were lower in the self-pollinated inflorescences in UR and JO populations (Fig. 4B and C). On the other hand, spontaneous selfing was comparable with cross-pollination in terms of seed number and seed-set rate. Since donor pollen from only one flower of the same ramet was used for each self-pollination treatment, the germination capacity of the pollen used might be directly related to the freshness of the flowers from which it was collected. Based on the result of the spontaneous-selfing experiment, self-fertilization did not reduce seed-set in the low-elevation populations.

It is common knowledge that populations maintained by selfing tend to have less attractive floral traits, such as smaller corolla size and reduced investment in male function (Sicard and Lenhard 2011). In our measurements, flower number per shoot in the self-compatible low-elevation populations was comparable to the self-incompatible alpine populations, and corolla size was even greater in the low-elevation populations. Therefore, the variation in floral morphology of *V. vitis-idaea* does not reflect the mating system of individual populations. Large flower size in the low-elevation habitats may be related to milder environmental conditions compared to alpine environment. Because outcrossing rate was maintained at least 50 % even in the self-compatible populations, the low-elevation populations may be maintained by a mixed-mating system of outcrossing and selfing. *Vaccinium vitis-idaea* is a long-lived clonal plant, and we detected the existence of huge clones (>30 m patch size) in several populations. Therefore, the extent to which seed production by selfing contributes to the maintenance of populations at low elevations is not clear.

### Variations in vegetative traits

The tetraploid populations were characterized by longer shoot length and larger leaves than the diploid populations. This may be partially due to the effect of whole-genome duplication. Polyploid individuals tend to have larger morphology and greater growth activity than diploids (Otto 2007) because of their larger cell size resulting from their increased DNA content (Ramsey and Ramsey 2014). Therefore, tetraploids may have high potential for vegetative growth and morphological regulation in response to the growth environments. In addition, morphological characteristics tended to be larger in the low-elevation populations than in the alpine populations, especially in the montane-forest habitat. Additionally, LMA was smaller in the montane-forest habitat and the coastal habitat. This variation probably reflected the light environment of each habitat. Alpine populations are basically located in open environments, whereas vegetation structure in the low-elevation habitats is more developed and complicated. Because understory of montane-forests is commonly shaded by the canopy of conifers (*Abies* and *Picea* trees), tall stature and large leaf size with lower LMA may be advantageous for light capture (Poorter *et al.* 2009). However, additional experimental approaches, such as common garden and transplant experiments, are required to discriminate the effects of ploidy level and habitat type on ecological characteristics, regarding that some plant species possess the quite large morphological plasticity regardless of the ploidy level (Mráz *et al.* 2012; Rosche *et al.* 2018).

## Conclusion

In summary, we discovered tetraploid populations of *V. vitis-idaea* for the first time; populations below the treeline are commonly tetraploids, whereas most of the alpine populations are diploids. Because tetraploid populations growing at low elevations are genetically and ecologically unique ecotypes different from typical alpine populations, they are valuable in terms of evolutionary and conservation biology. In future studies, a wider range of analyses of genotypes and ploidy levels are crucial in this species. Especially, comparisons with previous reports (Landolt 1996; Ikeda *et al.* 2015) are effective to clarify the origin and distribution range of tetraploid *V. vitis-idaea* at a geographic scale. In addition, it is important to understand the evolutionary significance of current genetic and ecological variation. Pollination experiments between different ploidy levels and between habitat types are needed to clarify the possibility of physiological mechanism of reproductive isolation.

## Supporting Information

The following additional information is available in the online version of this article—

**Figure S1.** Result of discriminant analysis of principal components (DAPC) analysis within (A) diploid populations and (B) tetraploid populations in *Vaccinium vitis-idaea*.

**Figure S2.** Relationships between geographic distance and genetic distance conducted for (A) all populations, (B) diploid populations and (C) tetraploid populations.

**Figure S3.** Comparison of values of principal component analysis (PCA) axes among ploidy level and habitat type. (A) PC1 value compared by ploidy level, (B) PC2 value compared by ploidy level, (C) PC1 value compared by habitat type and (D) PC2 value compared by habitat type.

**Table S1.** Summary of the researched populations, sampling year and sample size for individual analyses.

**Table S2.** Results of ploidy estimation based on the flow-cytometry analysis.

**Table S3.** Details of generalized linear model (GLM) and *post hoc* Tukey’s test for the pollination experiment in five *Vaccinium vitis-idaea* populations.

**Table S4.** Details of generalized linear model (GLM) and *post hoc* Tukey’s test on the results of floral morphology measurements in five *Vaccinium vitis-idaea* populations.

**Table S5.** Results of the measurement of vegetative characteristics. Mean and standard error of each ploidy and habitat type, and correlations with the principal component analysis (PCA) axes.

**Table S6.** Documented chromosome numbers of *Vaccinium vitis-idaea*.

plab015_suppl_Supplementary_Materials

plab015_suppl_Supplementary_Data

## Data Availability

The data used in this study are available as **Supporting Information**.

## Notes

**Populations & Communities. Chief Editor:** Jean Burns
