# Supplementary material for: Ecotypic differentiation of a circumpolar Arctic-alpine species at mid-latitudes: variations in the ploidy level and reproductive system of Vaccinium vitis-idaea
Source: AoB Plants. 2021 Apr 8;13(3):plab015. doi: 10.1093/aobpla/plab015 (PMC8114225; doi:10.1093/aobpla/plab015)
Supplement: plab015_suppl_Supplementary_Materials [file plab015_suppl_supplementary_materials.pdf]

Supporting Information

Figure S1. Discriminant Analysis of Principal Components (DAPC) within (A) diploid populations and (B) tetraploid populations in *Vaccinium vitis-idaea*. Colors indicate habitat type. Red: alpine; purple: open montane; green: montane forest; blue: coastal. Population codes follow those indicated in Table 1. The top right graph illustrates the principal component analysis and bottom right graph illustrates the eigen values of the discriminant analysis.

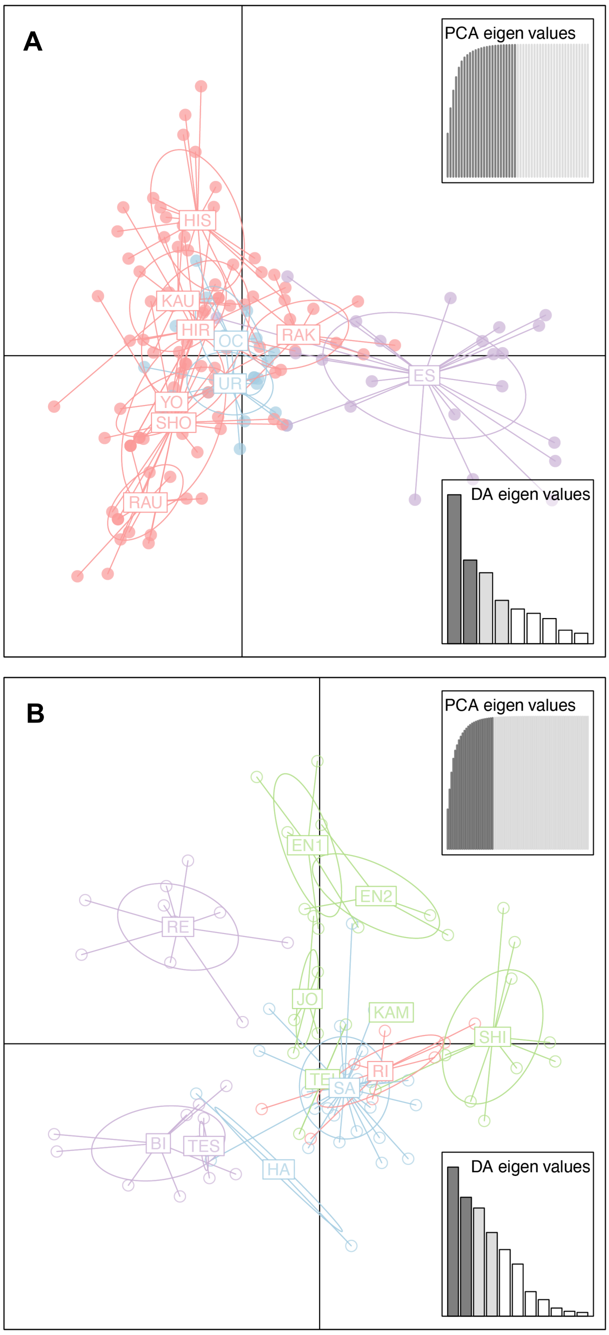

Figure S2. Isolation by distance analyses conducted for all populations (A), diploid populations (B), and tetraploid populations (C). Mantel test correlation values and their significance between geographic distances considering elevation differences and genetic distances (*Rho* values, Ronfort *et al.* 1998) are indicated for each analysis.

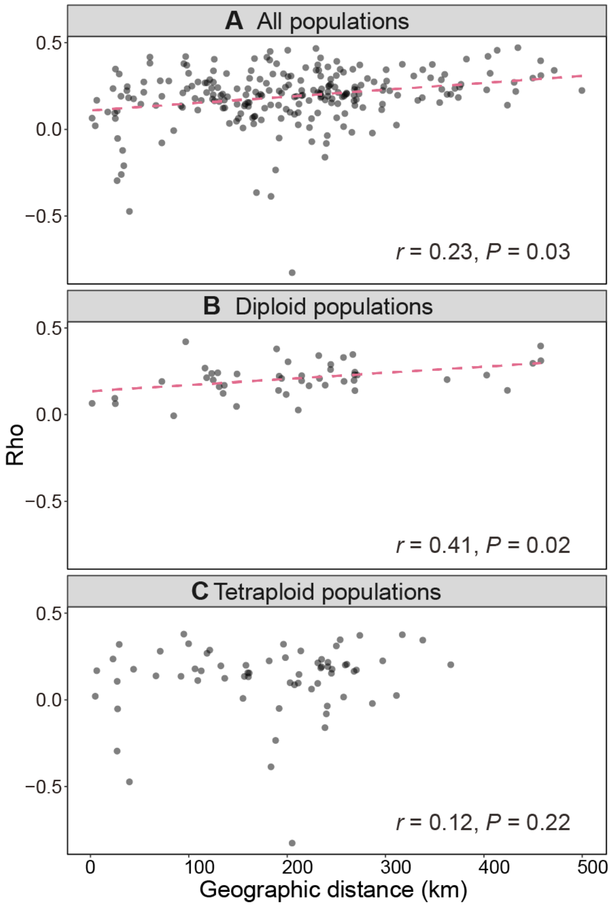

Figure S3. Comparison of values of PCA axes among ploidy level and habitat type. (A) PC1 value compared by ploidy level, (B) PC2 value compared by ploidy level, (C) PC1 value compared by habitat type and (D) PC2 value compared by habitat type.
Different letters represent significant differences ($P < 0.05$) according to ANOVA and Tukey's post-hoc test.

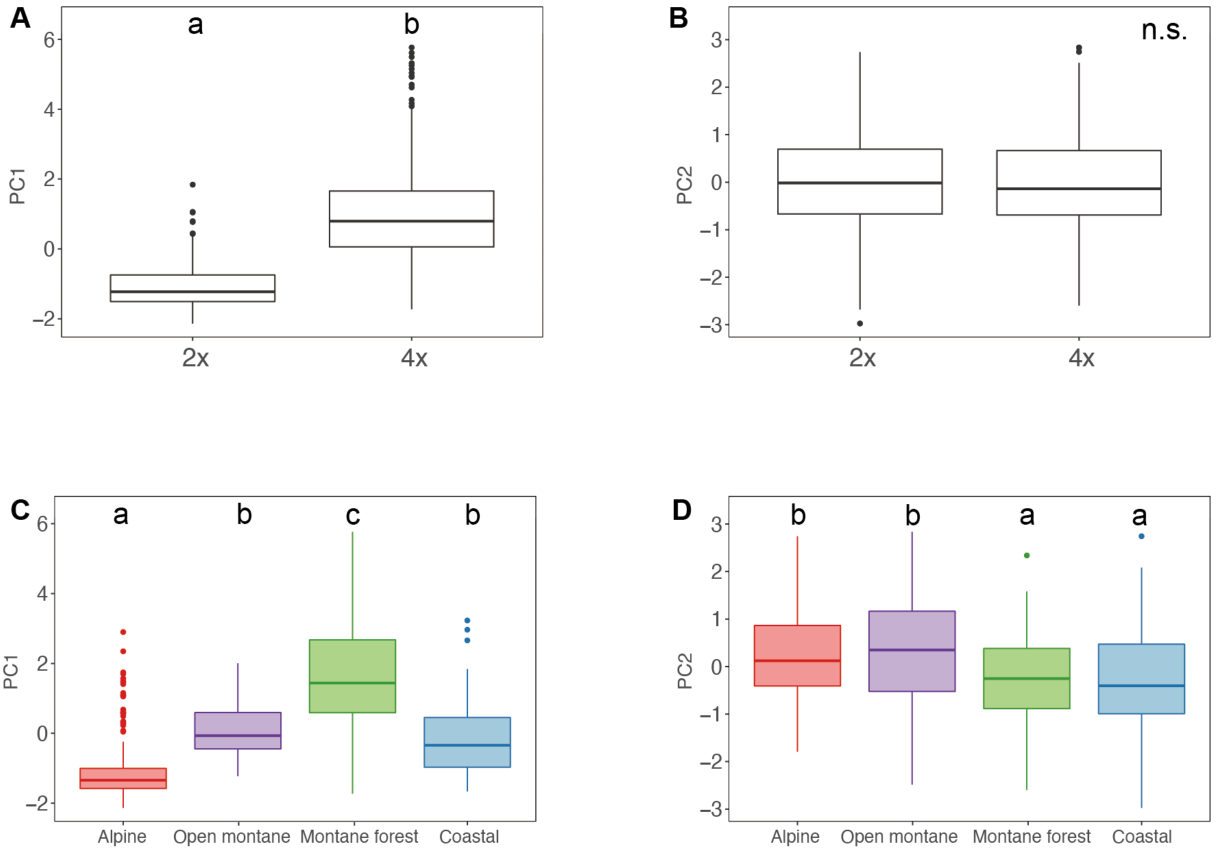

Table S1. Sampling number and year of all analysis. Individuals used for genetic analysis, survey of reproductive traits, pollination experiment and measurement of morphological traits were selected separately in the timing of each survey.

ID	Ploidy	Habitat type	Flow cytometry	Genetic analysis			Reproductive traits (Flower number, Fruit-set)		Reproductive traits (Seed number)		Pollination experiment (Fruit-set rate, Seed number, Seed-set rate, Seed weight)			Flower morph (Corolla size, Pistil length, Stamen length, ovule number)		Morphological traits (Annual shoot elongation, Leaf area, LMA)	
			N	Year	N (Leaves)	N (Seeds)	Year	N (Shoots)	Year	N (Fruits)	Year	N (Control indiv.)	N (Treated indiv.)	Year	N (Flowers)	Year	N (Shoots)
KAU	2x	Alpine	12	2016	30	73	2016	50	2016	25	2018	50	30	2018	30	2017	30
HIS	2x	Alpine	1	2016	30	74	2016	50	2016	25	–	–	–	–	–	2018	30
HIR	2x	Alpine	4	2016	30	69	2016	50	2016	24	2018	50	30	2018	30	2018	30
YO	2x	Alpine	3	2017	25	73	2017	50	2017	25	–	–	–	–	–	2017	30
RAU	2x	Alpine	2	2017	25	73	2017	50	2017	25	–	–	–	–	–	2017	30
SHO	2x	Alpine	3	2019	30	14	–	–	–	–	–	–	–	–	–	2019	30
RAK	2x	Alpine	3	2019	30	14	–	–	–	–	–	–	–	–	–	2019	30
ES	2x	Open montane	3	2018	30	53	–	–	–	–	–	–	–	–	–	2018	30
UR	2x	Coastal	6	2106	30	74	2016	50	2016	25	2018	50	30	2018	30	2017	30
OC	2x	Coastal	3	2017	25	55	2017	50	2017	22	–	–	–	–	–	2018	30
RI	4x	Alpine	3	2019	30	24	–	–	–	–	–	–	–	–	–	2019	30
BI	4x	Open montane	4	2017	25	51	2017	50	2017	22	–	–	–	–	–	2017	30
TES	4x	Open montane	7	2018	30	–	–	–	–	–	–	–	–	–	–	2018	30
RE	4x	Open montane	3	2019	30	16	–	–	–	–	–	–	–	–	–	2019	30
EN1	4x	Montane forest	3	2016	25	70	2016	50	2016	25	2018	50	30	2018	30	2017	30
EN2	4x	Montane forest	3	2016	25	66	2016	50	2016	25	–	–	–	–	–	2017	30
SHI	4x	Montane forest	4	2016	25	42	2016	50	2016	19	–	–	–	–	–	2019	30
JO	4x	Montane forest	4	2016	25	50	2016	50	2016	18	2018	50	30	2018	30	2017	30
TEI	4x	Montane forest	4	2016	25	66	2016	40	2016	24	–	–	–	–	–	2017	30
KAM	4x	Montane forest	–	2018	20	–	–	–	–	–	–	–	–	–	–	2018	30
HA	4x	Coastal	1	2016	30	57	2016	50	2016	23	–	–	–	–	–	2017	30
SA	4x	Coastal	4	2016	30	57	2016	50	2016	23	–	–	–	–	–	2018	30

Table S2. Summary of ploidy estimation using flow-cytometry.

Date	Analysis ID	Gain	Total count (tne number of nuclei analyzed)	Sample ID	Population	Value of relative fluorescence intensity	Coefficient of variation (CV)	Relative fluorescence intensity in target sample/standard sample	Estimated ploidy
2019/5/27	27708	379.5	4399	KAU-1	KAU	50.84	2.46	standard sample (=1.00)	2x = 24
2019/5/27	27709	379.5	1405	UR-1	UR	48.69	2.57	0.96	2x = 24
2019/5/27	27710	379.5	1350	UR-2	UR	48.39	2.58	0.95	2x = 24
2019/5/27	27711	379.5	1600	HIR-1	HIR	48.31	3.62	0.95	2x = 24
2019/5/27	27712	379.5	1336	HIR-2	HIR	47.95	2.61	0.94	2x = 24
2019/5/27	27713	379.5	1772	OC-1	OC	48.12	3.64	0.95	2x = 24
2019/5/27	27715	379.5	1662	ES-1	ES	48.53	3.61	0.95	2x = 24
2019/5/27	27717	379.5	1700	YO-1	YO	47.37	3.69	0.93	2x = 24
2019/5/27	27725	379.5	4374	JO-1	JO	100.53	1.74	1.98	4x = 48
2019/5/27	27727	379.5	2491	EN1-1	EN1	96.28	1.82	1.89	4x = 48
2019/5/27	27728	379.5	1196	EN1-2	EN1	95.35	1.31	1.88	4x = 48
2019/5/27	27729	379.5	1153	BI-1	BI	94.59	2.38	1.86	4x = 48
2019/5/27	27733	379.5	2167	KAU-2	KAU	44.9	2.78	0.88	2x = 24
2019/5/27	27733	379.5	2167	EN1-3	EN1	97.32	2.31	1.91	4x = 48
2019/9/21	28847	381.5	1184	KAU-3	KAU	48.45	2.58	standard sample (=1.00)	2x = 24
2019/9/21	28848	381.5	1002	HIS-1	HIS	49	2.55	1.01	2x = 24
2019/9/21	28845	381.5	1554	HIR-3	HIR	49.33	1.52	1.02	2x = 24
2019/9/21	28846	381.5	925	HIR-4	HIR	48.39	3.62	1.00	2x = 24
2019/9/21	28827	381.5	833	RAU-1	RAU	51.88	3.37	1.07	2x = 24
2019/9/21	28828	381.5	1414	RAU-2	RAU	51.73	2.42	1.07	2x = 24
2019/9/21	28834	381.5	1545	ES-2	ES	50.69	2.47	1.05	2x = 24
2019/9/21	28835	381.5	1782	ES-3	ES	50.43	2.48	1.04	2x = 24

2019/9/21	28825	381.5	1239	UR-3	UR	52.85	2.37	1.09	2x = 24
2019/9/21	28826	381.5	1390	UR-4	UR	51.75	2.42	1.07	2x = 24
2019/9/21	28849	381.5	1959	OC-2	OC	47	3.74	0.97	2x = 24
2019/9/21	28850	381.5	1868	OC-3	OC	48.29	2.59	1.00	2x = 24
2019/9/21	28860	381.5	2783	KAU-4	KAU	48.61	4.63	1.00	2x = 24
2019/9/21	28860	381.5	2783	BI-2	BI	97.78	1.79	2.02	4x = 48
2019/9/21	28843	381.5	929	BI-3	BI	104.2	1.68	2.15	4x = 48
2019/9/21	28844	381.5	1487	BI-4	BI	101.69	2.21	2.10	4x = 48
2019/9/21	28858	381.5	2518	KAU-5	KAU	47.55	3.68	0.98	2x = 24
2019/9/21	28858	381.5	2518	EN2-1	EN2	101.98	1.72	2.10	4x = 48
2019/9/21	28839	381.5	2763	EN2-2	EN2	102.42	2.2	2.11	4x = 48
2019/9/21	28840	381.5	1356	EN2-3	EN2	105.71	2.6	2.18	4x = 48
2019/9/21	28855	381.5	2134	KAU-6	KAU	47.83	2.61	0.99	2x = 24
2019/9/21	28855	381.5	2134	JO-2	JO	102.65	1.7	2.12	4x = 48
2019/9/21	28841	381.5	1853	JO-3	JO	103.5	2.17	2.14	4x = 48
2019/9/21	28842	381.5	1774	JO-4	JO	103.41	1.69	2.13	4x = 48
2019/9/21	28829	381.5	2970	SA-1	SA	105.16	2.14	2.17	4x = 48
2019/9/21	28857	381.5	1068	KAU-7	KAU	48.5	3.61	1.00	2x = 24
2019/9/21	28857	381.5	1068	TEI-1	TEI	100.18	2.25	2.07	4x = 48
2019/9/21	28832	381.5	1045	TEI-2	TEI	105.3	1.66	2.17	4x = 48
2019/9/21	28833	381.5	1110	TEI-3	TEI	106.13	2.12	2.19	4x = 48
2019/9/21	28838	381.5	1976	TEI-4	TEI	99.69	2.26	2.06	4x = 48
2019/9/21	28859	381.5	1759	KAU-8	KAU	48.22	3.63	1.00	2x = 24
2019/9/21	28859	381.5	1759	SA-2	SA	100.07	2.25	2.07	4x = 48
2019/9/21	28830	381.5	1135	SA-3	SA	105.99	2.12	2.19	4x = 48
2019/9/21	28831	381.5	1564	SA-4	SA	105.68	2.13	2.18	4x = 48

2019/9/30	29084	381	1060	UR-5	UR	50.98	2.45	standard sample (=1.00)	2x = 24
2019/9/30	29090	381	4304	UR-6	UR	47.86	4.7	0.94	2x = 24
2019/9/30	29090	381	4304	TES-1	TES	99.53	1.76	1.95	4x = 48
2019/9/30	29085	381	1064	TES-2	TES	103.37	2.18	2.03	4x = 48
2019/9/30	29086	381	2337	TES-3	TES	102.82	2.67	2.02	4x = 48
2019/9/30	29087	381	1179	TES-4	TES	103.16	1.21	2.02	4x = 48
2019/9/30	29088	381	1491	TES-5	TES	101.89	1.72	2.00	4x = 48
2019/9/30	29089	381	1745	TES-6	TES	101.57	1.72	1.99	4x = 48
2019/9/30	29091	381	4072	TES-7	TES	96.1	1.82	1.89	4x = 48
2019/11/19	29776	380	2791	KAU-9	KAU	51.76	2.42	standard sample (=1.00)	2x = 24
2019/11/19	29815	380	3579	YO-2	YO	50.96	2.45	0.98	2x = 24
2019/11/19	29816	380	2806	YO-3	YO	49.61	2.52	0.96	2x = 24
2019/11/19	29792	380	2806	SHO-1	SHO	50.75	3.45	0.98	2x = 24
2019/11/19	29793	380	3041	SHO-2	SHO	50.84	3.44	0.98	2x = 24
2019/11/19	29794	380	2256	SHO-3	SHO	50.9	2.46	0.98	2x = 24
2019/11/19	29786	380	2812	RAK-1	RAK	49.04	2.55	0.95	2x = 24
2019/11/19	29787	380	3363	RAK-2	RAK	49.62	2.52	0.96	2x = 24
2019/11/19	29788	380	2490	RAK-3	RAK	50.61	4.45	0.98	2x = 24
2019/11/19	29797	380	4956	KAU-10	KAU	50.56	3.46	0.98	2x = 24
2019/11/19	29797	380	4956	RI-1	RI	105.79	1.65	2.04	4x = 48
2019/11/19	29795	380	3370	RI-2	RI	103.27	2.66	2.00	4x = 48
2019/11/19	29796	380	3636	RI-3	RI	103.41	1.69	2.00	4x = 48
2019/11/19	29805	380	3115	KAU-11	KAU	51.01	3.43	0.99	2x = 24
2019/11/19	29805	380	3115	RE-1	RE	103.54	1.69	2.00	4x = 48
2019/11/19	29803	380	4881	RE-2	RE	104.54	2.63	2.02	4x = 48
2019/11/19	29804	380	3709	RE-3	RE	104.85	2.15	2.03	4x = 48
2019/11/19	29821	380	4292	KAU-12	KAU	50.66	2.47	0.98	2x = 24
2019/11/19	29821	380	4292	SHI-1	SHI	103.69	1.21	2.00	4x = 48

2019/11/19	29800	380	3059	SHI-2	SHI	100.96	2.23	1.95	4x = 48
2019/11/19	29801	380	3513	SHI-3	SHI	103.68	3.13	2.00	4x = 48
2019/11/19	29820	380	3217	SHI-4	SHI	104.17	2.64	2.01	4x = 48
2019/11/19	29806	380	2196	HA-1	HA	104.18	3.6	2.01	4x = 48

Table S3. Details in the results of GLMs indicated in Fig. 4: comparisons of fruit-set rate, seed number per fruit, seed-set rate, and seed weight across four treatments (C: control, OUT: cross-pollination, SELF: self-pollination, SSELF: spontaneous-selfing) in five population (KAU: Alpine 2*x*, HIR: Alpine 2*x*, UR: Coastal 2*x*, EN1: Montane forest 4*x*, JO: Montane forest 4*x*). ***P < 0.001, **P < 0.01, *P < 0.05. Blank: no significance according to GLM and Tukey's post-hoc test.

Population	Treatment	Fruit-set rate					Seed number / fruit					Seed-set rate					Seed weight				
		Estimate	SE	*z* value	*P* value		Estimate	SE	*z* value	*P* value		Estimate	SE	*z* value	*P* value		Estimate	SE	*z* value	*P* value	
KAU	C - OUT	1	0.18	5.45	< 0.001	***	-0.58	0.11	-5.35	< 0.001	***	-0.66	0.13	-4.94	< 0.001	***	0.07	0.05	1.42	0.16	
	C - SELF	-1.57	0.6	-2.63	0.03	*															
	C - SSELF																				
	OUT - SELF	-2.57	0.59	-4.38	0	***															
	OUT - SSELF																				
	SELF - SSELF																				
HIR	C - OUT	1.62	0.43	3.75	< 0.001	***	-0.3	0.14	-2.15	0.03	*	0.1	0.21	0.48	0.63		-0.01	0.04	-0.35	0.72	
	C - SELF	-0.01	0.74	-0.01	1																
	C - SSELF	-0.76	0.72	-1.06	0.7																
	OUT - SELF	-1.63	0.67	-2.42	0.07	.															
	OUT - SSELF	-2.37	0.65	-3.66	0	**															
	SELF - SSELF	-0.75	0.88	-0.85	0.82																
UR	C - OUT	1.11	0.14	8.1	< 0.001	***	-0.13	0.08	-1.63	0.36		0.02	0.1	0.21	1		0.11	0.04	2.7	0.03	*
	C - SELF	0.18	0.19	0.94	0.78		-0.66	0.1	-6.48	< 0.001	***	-0.59	0.12	-4.92	< 0.001	***	0.05	0.04	1.34	0.54	
	C - SSELF	0.05	0.18	0.29	0.99		0.03	0.08	0.4	0.98		0.07	0.11	0.63	0.92		0.17	0.04	4.15	< 0.001	***
	OUT - SELF	-0.93	0.18	-5.27	< 0.001	***	-0.53	0.1	-5.29	< 0.001	***	-0.62	0.12	-4.94	< 0.001	***	-0.06	0.04	-1.37	0.52	
	OUT - SSELF	-1.06	0.17	-6.3	< 0.001	***	0.16	0.08	1.99	0.19		0.05	0.11	0.41	0.98		0.06	0.04	1.45	0.47	
	SELF - SSELF	-0.13	0.21	-0.6	0.93		0.69	0.1	6.69	< 0.001	***	0.66	0.13	5.24	< 0.001	***	0.11	0.04	2.81	0.03	*

EN1	C - OUT	1.71	0.32	5.32	< 0.001	***	0.15	0.16	0.94	0.78		0.4	0.19	2.05	0.17		-0.08	0.04	-2.02	0.18	
	C - SELF	1.42	0.36	3.91	< 0.001	***	-0.32	0.19	-1.66	0.34		-0.15	0.24	-0.65	0.91		0.03	0.04	0.84	0.84	
	C - SSELF	0.77	0.37	2.08	0.16		-0.22	0.19	-1.16	0.65		-0.07	0.24	-0.29	0.99		0.05	0.04	1.21	0.62	
	OUT - SELF	-0.29	0.31	-0.95	0.78		-0.47	0.21	-2.29	0.1	.	-0.55	0.26	-2.14	0.14		0.11	0.04	2.79	0.03	*
	OUT - SSELF	-0.95	0.31	-3.04	0.01	*	-0.38	0.21	-1.83	0.26		-0.47	0.26	-1.78	0.28		0.13	0.04	3.01	0.01	*
	SELF - SSELF	-0.66	0.36	-1.85	0.25		0.1	0.23	0.42	0.98		0.08	0.3	0.28	0.99		0.02	0.04	0.44	0.97	
JO	C - OUT	1.09	0.27	4.01	< 0.001	***	-0.45	0.16	-2.76	0.03	*	-0.23	0.19	-1.19	0.63		-0.05	0.04	-1.26	0.59	
	C - SELF	0.61	0.35	1.76	0.29		-1.05	0.2	-5.18	< 0.001	***	-0.78	0.24	-3.28	0.01	**	0	0.05	-0.08	1	
	C - SSELF	0.23	0.34	0.67	0.91		-0.32	0.17	-1.91	0.22		-0.21	0.19	-1.08	0.7		0	0.04	0.04	1	
	OUT - SELF	-0.48	0.32	-1.5	0.43		-0.61	0.23	-2.67	0.04	*	-0.56	0.27	-2.08	0.15		0.05	0.05	0.94	0.78	
	OUT - SSELF	-0.87	0.32	-2.74	0.03	*	0.13	0.2	0.64	0.92		0.02	0.23	0.07	1		0.05	0.04	1.29	0.57	
	SELF - SSELF	-0.38	0.38	-1	0.75		0.73	0.23	3.16	0.01	**	0.57	0.27	2.12	0.14		0.01	0.05	0.11	1	

Table S4. Details in the result of GLMs indicated in Fig. 5: comparison of floral morphology among five populations (KAU: Alpine 2*x*, HIR: Alpine 2*x*, UR: Coastal 2*x*, EN1: Montane forest 4*x*, JO: Montane forest 4*x*) based on GLM and Tukey's post-hoc test. ***$P < 0.001$, **$P < 0.01$, *$P < 0.05$. Blank: no significance according to GLM and Tukey's post-hoc test.

		Estimate	SE	*z* value	*P* value	
Corolla size	KAU-HIR	2.791	3.08	0.906	0.895	
	KAU-UR	47.452	3.08	15.406	<0.001	***
	KAU-EN1	42.085	3.08	13.664	<0.001	***
	KAU-JO	19.362	3.08	6.286	<0.001	***
	HIR-UR	44.66	3.08	14.5	<0.001	***
	HIR-EN1	39.294	3.08	12.758	<0.001	***
	HIR-JO	16.571	3.08	5.38	<0.001	***
	UR-EN1	-5.366	3.08	-1.742	0.408	
	UR-JO	-28.09	3.08	-9.12	<0.001	***
	EN1-JO	-22.723	3.08	-7.378	<0.001	***
Pistil length	KAU-HIR	-0.272	0.032	-8.563	<0.001	***
	KAU-UR	0.278	0.032	8.761	<0.001	***
	KAU-EN1	0.325	0.032	10.225	<0.001	***
	KAU-JO	0.373	0.032	11.751	<0.001	***
	HIR-UR	0.551	0.032	17.324	<0.001	***
	HIR-EN1	0.597	0.032	18.787	<0.001	***
	HIR-JO	0.646	0.032	20.314	<0.001	***
	UR-EN1	0.047	0.032	1.464	0.586	
	UR-JO	0.095	0.032	2.99	0.023	*
	EN1-JO	0.049	0.032	1.527	0.545	
Stamen length	KAU-HIR	-0.188	0.041	-4.631	<0.001	***
	KAU-UR	0.366	0.041	9.032	<0.001	***
	KAU-EN1	0.185	0.041	4.563	<0.001	***
	KAU-JO	0.369	0.041	9.101	<0.001	***
	HIR-UR	0.554	0.041	13.663	<0.001	***
	HIR-EN1	0.373	0.041	9.194	<0.001	***
	HIR-JO	0.557	0.041	13.731	<0.001	***
	UR-EN1	-0.181	0.041	-4.469	<0.001	***
	UR-JO	0.003	0.041	0.069	1	
	EN1-JO	0.184	0.041	4.537	<0.001	***

Ovule number / fruit	KAU-HIR	-0.221	0.094	-2.36	0.125	
	KAU-UR	0.315	0.078	4.056	<0.001	***
	KAU-EN1	-0.169	0.081	-2.082	0.226	
	KAU-JO	-0.071	0.081	-0.871	0.907	
	HIR-UR	0.536	0.091	5.911	<0.001	***
	HIR-EN1	0.052	0.094	0.557	0.981	
	HIR-JO	0.15	0.094	1.606	0.491	
	UR-EN1	-0.484	0.078	-6.23	<0.001	***
	UR-JO	-0.385	0.078	-4.965	<0.001	***
	EN1-JO	0.098	0.081	1.211	0.744	
Seed weight	KAU-HIR	0.097	0.039	2.523	0.085	.
	KAU-UR	0.292	0.039	7.574	< 0.001	***
	KAU-EN1	0.6	0.039	15.547	< 0.001	***
	KAU-JO	0.462	0.039	11.979	< 0.001	***
	HIR-UR	0.195	0.039	5.051	< 0.001	***
	HIR-EN1	0.503	0.039	13.024	< 0.001	***
	HIR-JO	0.365	0.039	9.456	< 0.001	***
	UR-EN1	0.308	0.039	7.973	< 0.001	***
	UR-JO	0.17	0.039	4.405	< 0.001	***
	EN1-JO	-0.138	0.039	-3.568	0.003	**

Table S5. Details of the measurement of vegetative characteristics. Mean and standard error within each ploidy and habitat type, and Pearson's correlation coefficients (r) with the axes of PCA analysis. ***$P < 0.001$, **$P < 0.01$, *$P < 0.05$. Blank: no significance

Trait	Ploidy		Habitat type				PC1			PC2		
	2x	4x	Alpine	Open montane	Montene forest	Coastal	r	P		r	P	
Annual shoot elongation (mm)	18.73 ± 8.74	34.76 ± 13.39	16.18 ± 8.06	28.12 ± 9.46	40.65 ± 13.56	27.93 ± 9.72	0.75	< 0.001	***	-0.16	< 0.001	***
Leaf area (cm^2)	0.6 ± 0.21	1.51 ± 0.7	0.64 ± 0.35	1.05 ± 0.34	1.81 ± 0.83	0.92 ± 0.43	0.96	< 0.001	***	0.03	0.5	
Leaf mass (mg)	8.77 ± 3.69	19.42 ± 9.05	9.81 ± 5.75	15.91 ± 6.63	20.73 ± 10.66	12.64 ± 7.06	0.90	< 0.001	***	0.35	< 0.001	***
LMA (g/m^2)	145.5 ± 29.7	137.7 ± 29.2	150.8 ± 26.6	149.5 ± 30	128.7 ± 27.3	134.3 ± 29.6	-0.24	< 0.001	***	0.95	< 0.001	***

Table S6. Documented chromosome numbers of *Vaccinium vitis-idaea*

Species	Chromosome number	References
Vaccinium vitis-idaea L.	2*n* = 24	Hagerup (1928)*, Tischler (1934)**, Rohweder (1937)*, Newcomer (1941)*, Löve (1954), Löve & Löve (1956)*, Sorsa (1962)*, Hedberg & Hedberg (1964), Rousi (1966)*, Rousi (1967)*, Packer & McPherson (1974)*, Uhrikova *et al.* (1980)*, Majovsky & Uhrikova (1982)*, Arohonka (1982)*, Löve & Löve (1982)**, Dmitrieva (1985c)*, Zhukova & Petrovsky (1987), Měsíček (1992)**, Li *et al.* (1993), Chen (1993)*, Druskovic & Lovka (1995)*, Lövkvist & Hultgård (1999)**
	n = 12	Rohweder (1937)*, Laane (1965), Rousi (1966)*, Rousi (1967)*
	n = ca. 12	Sorsa (1962)*
	n = 12, 14-22<br>*n* = 34, 36	Ahokas (1971), Uotila & Pellinen (1985)*
ssp. *minus* Hulten	2*n* = 24	Löve & Löve (1966)*, Sokolovskaya (1968), Zhukova *et al.* (1977), Johnson & Packer (1968), Löve & Löve (1982)**, Landolt (1996)
	n =12	Taylor & Mulligan (1968)*, Pojar (1973)*, Dalgaard (1989)

* Nishikawa (2008), ** Rice *et al.* (2014)

References

Löve A. 1954 Cytotaxonomical evaluation of corresponding taxa. *Vegetatio* 5-6: 212-224

Hedberg I. & Hedberg O. 1964 Documented chromosome numbers of Swedish plants. *Svensk Bot. Tidskr.* 58: 125-128

Zhukova P.G. & Petrovsky V.V. 1987 Chromosome numbers and taxonomy of some plant species from the northern Asia regions Bot. Zhurn. 72: 1617-1624

Li, X.-l. et al. 1993 Studies on karyotype of some berry plants in north China. J. *Wuhan Bot. Res.* 11(4): 289-292

Laane M. M. 1965 Kromosomundersokelser hos noen norske planter. Blyttia 23: 169-189

Ahokas H. 1971. Notes on polyploidy and hybridity in *Vaccinium* species. Annales Botanici Fennici 8: 254–256.

Sokolovskaya A. P. 1968 Kariologicheskoe issledovanie flory Koryatskoy zemli. *Bot. Zhurn.*

53:99-105
Zhukova P. G. et al. 1977 Numbers of some plant species in the eastern arctic Jakutia. *Bot. Zhurn.* 62: 228-234
Johnson A. W. & Packer J. G. 1968 Chromosome numbers in flora of Ogotruk Creek, N.W Alaska. Bot. Not.121: 403-456.
Landolt E. 1996. *Vaccinium vitis-idaea* L. subsp. minus (G. Lodd.) Hultén (Ericaceae), an overlooked circumpolar-arctic taxon of the alps. Anales del Jardín Botánico de Madrid 54: 277–284.
Dalgaard V.1989 Additional chromosome numbers in vascular plants from the Disko Bugt area (West. Greenland). Willdenowia 19: 199-213.
Nishikawa T. 2008. Chromosome atlas of flowering plants in Japan. National Museum of Nature and Science, Tokyo, Japan
Rice A, Glick L, Abadi S, et al. 2015. The Chromosome Counts Database (CCDB) - a community resource of plant chromosome numbers. New Phytologist 206: 19–26.
